# A Hybrid {Silk@Zirconium MOF} Material as Highly Efficient As^III^-sponge

**DOI:** 10.1038/s41598-020-66091-w

**Published:** 2020-06-09

**Authors:** Yiannis Georgiou, Sofia Rapti, Alexandra Mavrogiorgou, Gerasimos Armatas, Manolis J. Manos, Maria Louloudi, Yiannis Deligiannakis

**Affiliations:** 1 Laboratory of Biomimetic Catalysis and Hybrid Materials, Department of Chemistry, University of Ioannina, GR45110 Ioannina, Greece; 2 Laboratory of Physical Chemistry of Materials and Environment, Department of Physics, University of Ioannina, GR45110 Ioannina, Greece; 3 Department of Materials Science and Technology, University of Crete, Heraklion, 71003 Greece; 4 Institute of Materials Science and Computing, University Research Center of Ioannina, GR45110 Ioannina, Greece; 5 Institute of Environment & Sustainable Development, University Research Center of Ioannina, GR45110 Ioannina, Greece

**Keywords:** Environmental chemistry, Pollution remediation

## Abstract

Exposure of humans to Arsenic from groundwater drinking sources is an acute global public health problem, entailing the urgent need for highly efficient/low-cost Arsenite (As^III^) up-taking materials. Herein we present an innovative hybrid-material, ZrMO*F@SF*_*d*_ operating like an “As^III^-sponge” with unprecedented efficiency of 1800 mg As^III^ gr^−1^. ZrMOF@*SF*_*d*_ consists of a neutral Zirconium Metal-Organic Framework [ZrMOF] covalently grafted on a natural silk-fiber (*SF*_*d*_). ZrMOF itself exhibits As^III^ adsorption of 2200 mg gr^−1^, which supersedes any -so far- known As^ΙΙΙ^-sorbent. Using XPS, FTIR, BET-porosimetry data, together with theoretical Surface-Complexation-Modeling (SCM), we show that the high-As^ΙΙΙ^-uptake is due to a sequence of two phenomena:[i] at low As^III^-concentrations, surface-complexation of H_3_AsO_3_ results in As^III^-coated voids of ZrMOF, [ii] at increased As^III^-concentrations, the As^III^-coated voids of ZrMOF are filled-up by H_3_AsO_3_ *via* a partitioning-like mechanism. In a more general context, the present research exemplifies a mind-changing concept, i.e. that a “partitioning-like” mechanism can be operating for adsorption of metalloids, such as H_3_AsO_3,_ by metal oxide materials. So far, such a mechanism has been conceptualized only for the uptake of non-polar organics by natural organic matter or synthetic polymers.

## Introduction

Arsenic exposure through drinking water sourced from groundwater, is a global public health problem that is particularly devastating in certain highly populated countries^1,2^. According to a 2000 to 2010 case study, 35 to 77 million people in areas of Bangladesh or India have been chronically exposed to arsenic in their drinking water in what described as the most significant mass poisoning in history^3^. Arsenic is a naturally occurring metalloid, also released to the environment *via* anthropogenic activities. Arsenic strongly binds to proteins, so traces of this element can cause severe health problems to all life forms^4^. The predominant forms of arsenic in the aquatic environment are As^III^ (arsenite) and As^V^ (arsenate). As^III^ is more hazardous than As^V^, as it is more mobile/bioavailable, thus more toxic^5^. This toxicity is due to its dominant H_3_AsO_3_ form, i.e. the predominating species in a wide range of pH < 9^6^, typically encountered in natural waters. So far, traditional ion-exchange materials and sorbents, e.g. zeolites, clays, layered double hydroxides, resins have been used as arsenic adsorbends, however, with limited efficiency *vs*. As^III^ ^7,8^.

Given that As^V^, which exists as anion at pH > 2, is easier to adsorb on cationic surfaces, to overcome the low efficiency of As^III^ uptake, an extra step of oxidation of As^III^ to As^V^ can be chosen before the application of various remediation technologies. Such oxidative pretreatment, however, suffers from the presence of multiple substances that interfere with As^III^ oxidation^9,10^. Therefore, it will be desirable to develop sorbents that could *directly* capture As^III^ in natural pH conditions, without the need for the oxidation to As^V^. Several materials have been investigated for the direct removal of As^III^, including TiO_2_ nanoparticles^11^, iron-based nanoparticles, e.g. zero-valent Fe nanoparticles^12–14^, carbon nanotubes^15^. Such sorbents with high specific surface area and various functional groups seem promising for As^III^ remediation^16–18^.

A further hurdle to overcome concerns cost-criteria, i.e. the required mass of an As^III^-uptaking material, should be considered on a cost-efficiency basis together with the ecological impact of such sorbents is natural or urban water bodies. So far, most of the available materials have sorption capacities of the range 60–150 mg g^−1^ ^19–21^, while less than five materials achieving sorption capacities > 300 mg g^−1^ ^22,23^. In one report in 2014^23^, the highest –so far- As^III^ uptake reported was 320 mg gr^−1^ by a hybrid consisting of Fe_2_O_3_ nanoparticles dispersed on a macroporous silica. Our systematic efforts during the last decade led to a series of nanomaterials with promising performances, e.g. a mesoporous spinel CoFe_2_O_4_^24^ with an uptake of 252 mg As^III^ g^−1^, magnetic carbon nanocages^22^ with a sorption capacity of 264 mg As^III^ g^−1^ and MIL-100(Fe)^16^ showing uptake of 120 mg As^III^ g^−1^.

Regarding the physicochemical As^III^-uptake mechanism, so far, in all well-understood cases, the underlying theoretical mechanism is that originally developed by Goldberg^25,26^ and Manning^7,10^, which entails that: [i] As^III^-uptake by solid materials is determined by surface complexation of the As^III^ species. [ii] at pH range 5–8, i.e. typical for natural waters, the dominant species is the neutral form H_3_AsO_3_^27,28^. Thus –so far – the strategy by all research groups, including us^16,22,29^, was to maximize the number and accessibility of surface sites based on diligent preparation protocols. In this way, it has been achieved a max As^III^-uptake capacity 320 mg gr^−1^ by *γ*-Fe_2_O_3_ nanoparticles encapsulated in a macroporous Silica^23^. Recent data show that certain carbon based materials i.e. graphene-based^30^ or more innovative graphydine^31–33^ have a promising potential for adsorbing heavy metals, metalloids and other pollutants from water. In addition, some of the metal-loaded materials can have enhanced catalytic fucntionalities^34–37^.

Within this frame of thinking, aiming at maximization of the surface sites, it becomes obvious that for any material, the theoretical upper limit would be determined by the site-density and the surface area:

1 $${N}_{max}(sites/gram)=SSA({m}^{2}/gr)\times Ns(sites/n{m}^{2})={10}^{18}\times SSA(n{m}^{2}/g{r}^{1})\times Ns(sites/n{m}^{2})$$

Using this expression, a material whose maximum- As^III^ uptake capacity is determined by surface-adsorption has an upper theoretical limit, which under ideal conditions, would be determined by its specific surface area (SSA) and the number of surface sites (Ns). In real systems, this maximum-uptake would be further limited by the binding constant of As^III^-on the surface-sites. On the other hand, in natural systems, Soil Organic Matter is known to be able to sequester non-polar organics *via* a partitioning-sequestration mechanism^38^. This phenomenon is based on the fundamental concept of the *partitioning* of a non-polar organic between a polar and a non-polar solvent, e.g. for example, the partitioning of phenol on [octanol: water]^39,40^. Within a technological context, when partitioning is operating, a significant mass of sorbent can be transferred in the apolar matrix, thus resulting in cost-effective molecular-separation approaches^41,42^. So far, however, such a profitable concept has not been applied to attack problems such as As^III^-remediation. Herein we introduce a mind-changing approach by developing a Zirconium Metal-Organic Framework [[Zr_6_O_4_(OH)_4_(NH_2_BDC)_6_] (NH_2_-BDC^2−^ = 2-amino-terephthalate) [herein codenamed ZrMOF] which is able to perform a partitioning-like As^III^-uptake thanks to the microenvironment of its pores. As we show, this approach allows an unprecedented As^III^-uptake efficiency of > 2000 mg As^III^ per gram of ZrMOF.

Metal-organic frameworks (MOFs), which are crystalline porous materials are constituted by metal ions or metal clusters, and polytopic organic ligands, have emerged as a new class of sorbents with a promise for various remediation processes^43–45^. MOFs combine extremely high surface areas, well-defined pores, and a variety of functional groups. Furthermore, several MOFs show remarkable thermal (up to 400–500 °C) and chemical stability e.g. high resistance to acid or base^46,47^. Recently, several MOFs have been investigated as arsenic sorbents, mostly concerning investigations of forms of ionic-As^V^ sorption^48–50^. Only a few reports exist on the capture of As^III^ by MOFs^40,43–45^. These sorbents, however, demonstrated only moderate sorption capacities (<150 mg As^III^ g^−1^)^51^.

Apart from the challenge of As^III^-uptake efficiency, the efficient large-scale utilization of As^III^-uptaking materials^52–54^, requires their post-synthesis engineering to be usable for large-water body cleaning. The technology of grafting of the functional material on a macroscopic surface allows scale-up handling and usage. Herein we have used woven silk fibers [SF_d_] as a scaffold for grafting of the ZrMOF material. The so-obtained hybrid material ZrMOF@*SF*_*d*_ retains the very high As^III^ sorption capacity of ZrMOF, i.e. reaching 1800 mg As^III^ per g^−1^ of material.

To understand the unprecedented high sorption efficiency of ZrMOF and ZrMOF@*SF*_*d*,_ we have carried out a detailed study ofthe As^III^-sorption mechanism in conjunction with the dynamics of pore-filling and surface complexation. In a more general context, the present research exemplifies for the first time that a “partitioning-like” mechanism to be operating in for adsorption of metalloids, i.e., H_3_AsO_3_ by metal oxide materials, so far conceptualized only for synthetic polymers & natural organic matter (NOM)^55^ used to uptake apolar organics^56,57^.

## Results and Discussion

**Figure 1:**
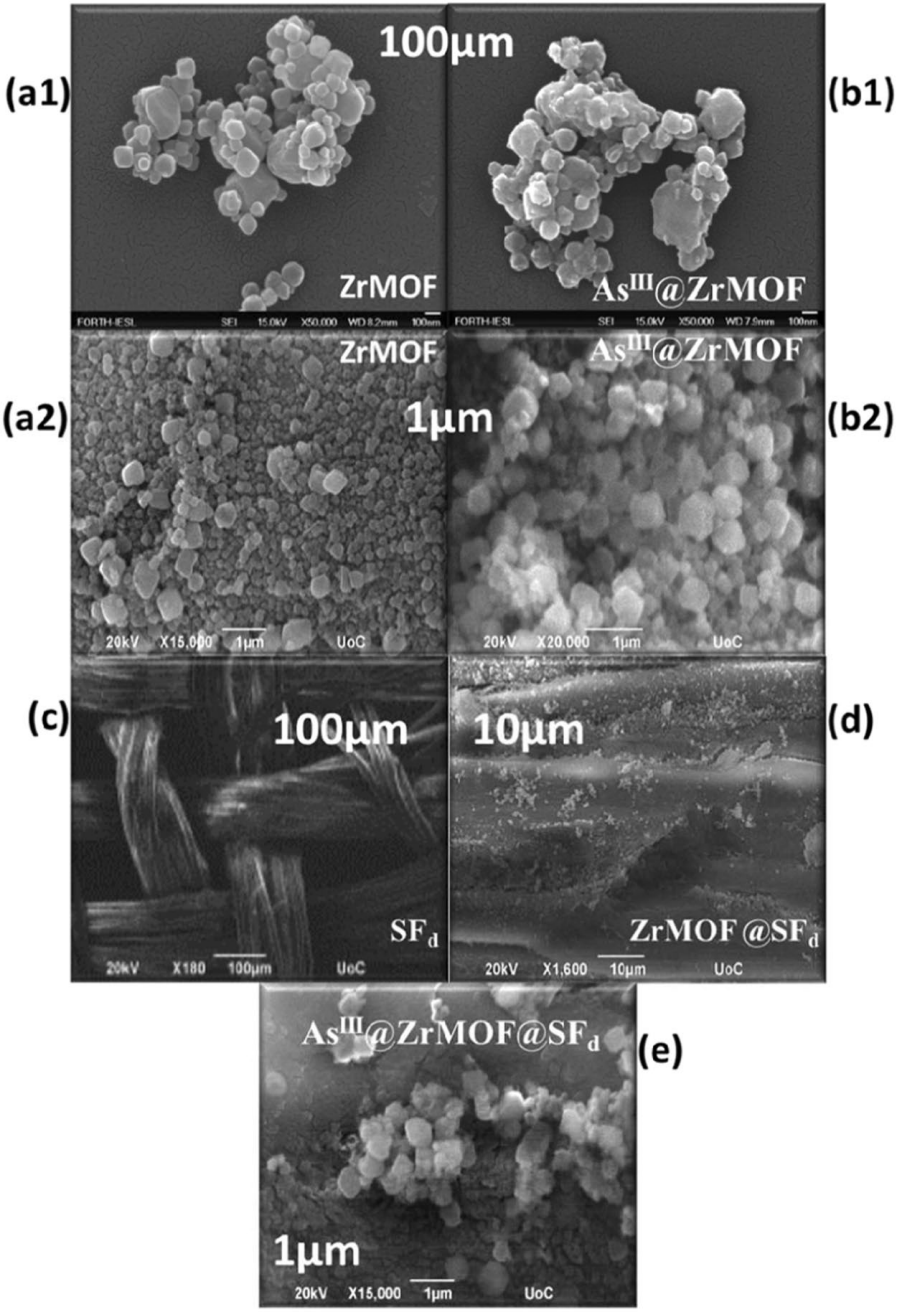
FE-SEM images for ZrMOF (**a1,a2**), As^III^-loaded ZrMOF (**b1,b2**), natural-silk SF_d_ (**c**),ZrMOF@SF_d_ (**d**), As^III^-loaded ZrMOF@SF_d_ (**e**).

Field Emission-Scanning electronic microscopy (FE-SEM) images showed that ZrMOF is composed of aggregated polyhedral-shape nanoparticles with size ~100–300 nm **(**Figure 1a1,a2). No obvious changes in shape and size of particles are observed for the material after the As^III^ sorption (Fig. 1b1,b2). The SEM micrograph for the silk fiber (SF_d_) (Fig. 1c), shows the surface morphology of well-defined fibers of natural-silk. After covalent grafting of ZrMOF on the SF_d_ fibers, we obtain well-dispersed ZrMOF particles on the silk-fibers (Fig. 1d). Adsorption of As^III^ onto ZrMOF@SF_d_ does not alter the particle morphology (Fig. 1e). Thermogravimetric (TGA) analysis, Fig. S1 in Supporting Information, shows that the ZrMOF@SF_d_ hybrid contains 5.7% w:w of ZrMOF.

**Figure 2:**
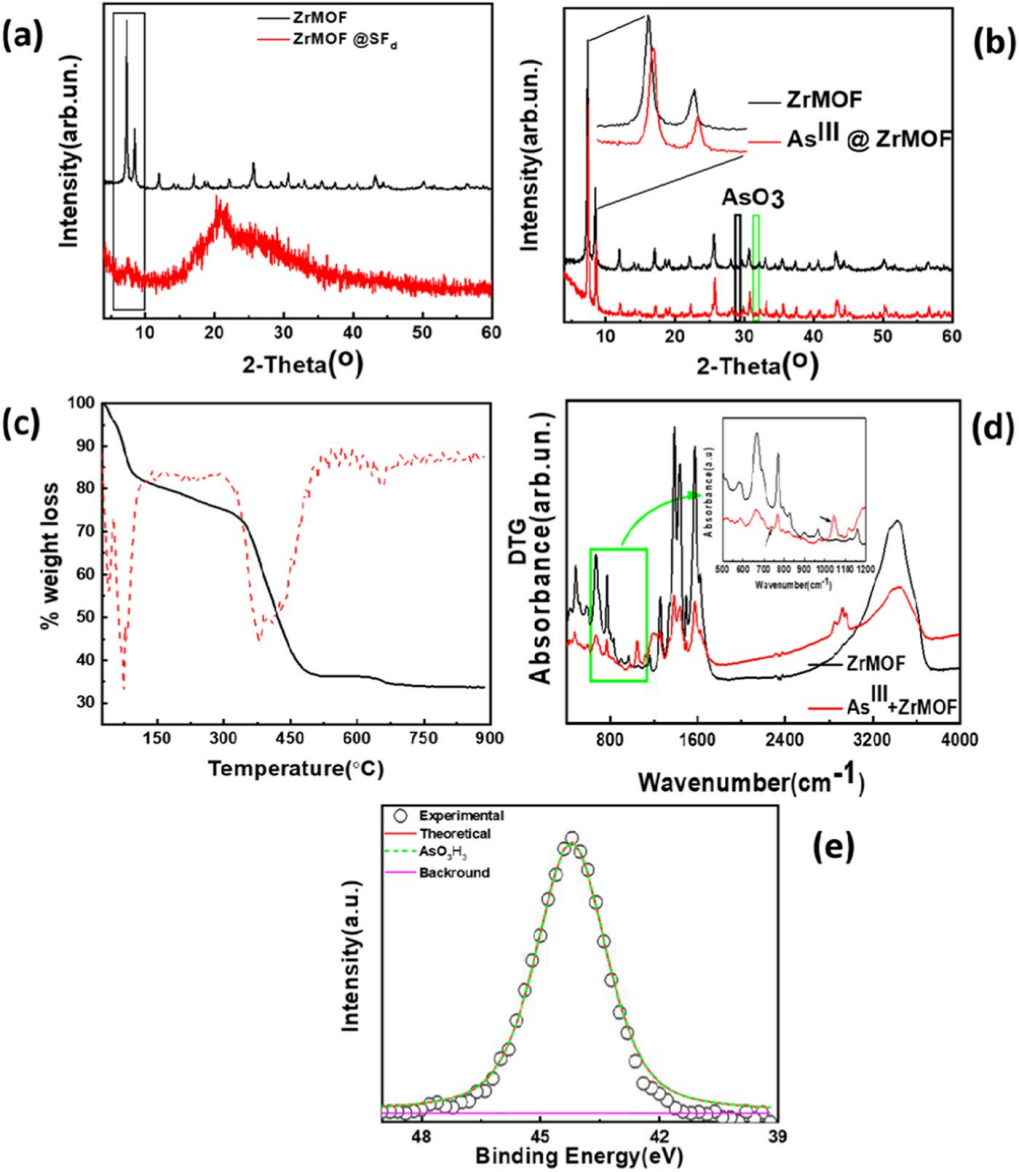
(**a**) PXRD data for ZrMOF and ZrMOF @ SF_d_, (**b**) PXRD data for ZrMOF, and As^III^@ ZrMOF. (**c**)TGA (solid line) and first derivative (DTG) (dashed line) plots for ZrMOF,(**d**) FT**-**IR spectra for pristine (**−**), and As^III^-loaded ZrMOF material (), (**e**) XPS As3d analysis for ZrMOF after As^III^ loading.

For the engineering of the ZrMOF grafting on the silk fiber, we have used natural silk tissue, which we have degummed according to established procedures (see Supporting Information, photos in Scheme S1)^58^. The degumming method of Gulrajani^58^, resulted in high-quality silk-fiber, as evidenced by the SEM micrography, see Fig. 1(c), as well as the XRD pattern, Fig. 2A, which reveals retention of the fibers’ order and physical integrity in the structure of the degummed silk (SF_d_). After grafting of the ZrMOF@SF_d,_ the SEM data (Fig. 1d) shows a good dispersion of the ZrMOF particles on the silk fibers. XRD data for the ZrMOF@SF_d_ hybrid, Fig. 2a(red) show the characteristic reflection at 7.3° and 8.5° originating from the ZrMOF particles grafted on the silk. Notice that, upon grafting, the crystallinity of the SF_d_ is distorted, i.e. see the loss of the sharp peaks at 15°–17° in Fig. 2a. This result is due to molecular covalent grafting of the ZrMOF-silane on SF_d_ (see also Scheme S2 in Supporting Information)

TGA analysis, Fig. 2c, revealed a significant weight loss (~17.2%) in the temperature range 25 to 102 °C attributed to the release of solvent molecules, mostly MeOH. Then, there is a continuous weight loss (~7.9%) (with no discrete steps) till 302 °C, followed by an abrupt weight loss (38.8%) ending at 540 °C. Finally, there is a small weight loss (~2%) from 615 to 715 °C (Fig. 2c). The TGA residue is solid ZrO_2,_ as confirmed by XRD data. For 100 g of ZrMOF 33.7 g of ZrO_2_ was obtained after calcination, which corresponds to ~24.9% Zr w:w. Based on this % Zr content found from TGA, the suggested formula for ZrMOF is [Zr_6_O_4_(OH)_4_(NH_2_-BDC)_6_]∙12MeOH ∙ 3H_2_O (calculated % Zr = 24.96).

The pristine silk fabric (SF) and degummed silk fibroin fibers (SF_d_) were characterized by FT-IR and thermogravimetric analysis (TG-DTA). The typical IR-peaks of SF are ≈1620–1700, 1511–1539, and 1226–1235 cm^−1^, characteristic for amide I (C=O stretching), amide II (N–H deformation, and C–N stretching) and amide III (C–N stretching and N–H deformation). FT-IR (cm^−1^, selected peaks) SF: 3533: ν(OH); 3072, 2980, 2936, 2880: ν(C-H); 1697: amide I (*β*-sheet); 1595: amide II (*β*-sheet); 1256: amide III (*β*-sheet), 1166: ν(C-ΟH). SF_d_ 3477: ν(OH); 3075, 2980, 2936, 2880: ν(C-H); 1708, amide I (*β*-sheet); 1595: amide II (β-sheet); 1271: amide III (*β*-sheet), 1001: ν(C-ΟH) (see Fig. S2). Both TG-DTA curves (see Supporting Information Fig. S1) for the SF and the SF_d_ show similar thermal-response behavior. At T < 110 °C the weight loss is attributed to the evaporation of water. The change from 170 °C to 275 °C can be assigned to the loss of other low-temperature volatile species, and the change from 275 °C to 400 °C is associated with the breakdown of side-chain groups of amino acid residues as well as the cleavage of peptide bonds of silk fiber, and at T > 400 °C it is attributed to fibroins’ degradation^59^. The TG% curves of SF and SF_d_ exhibit a total weight loss of 98.4% and 98.9% respectively, in the range 20–700 °C with a broad exothermic peak at 400–600 °C assigned to the fibroins’ degradation. The DTA curve of SF_d_ also shows an intense peak at 577 °C, originating from the amorphous sericin extraction^59^ and the degummed *β*-sheet fibroin degradation^59^.

The final ZrMOF@SF_d_ hybrid was characterized by FT-IR and thermogravimetric analysis (TG-DTA) (see Fig. S1 in Supporting Information). The typical IR-peaks of SF are ≈1620, 1511, and 1226 cm^−1^, characteristic for amide I (C=O stretching), amide II (N–H deformation, and C–N stretching), and amide III (C–N stretching and N–H deformation). FT-IR (cm^−1^, selected peaks) ZrMOF@SF_d_: ν(OH); ν(C-H); ν(C=C); ν(C-OH); ν(C-Ο) (see Fig. S1 in Supporting Information). The TG-DTA curves for ZrMOF@SF_d_ show a thermal-response profile similar to that of the SF_d_ fibers. The TG% curve of ZrMOF@SF_d_ exhibits a total weight loss of 97.3% in the range of 20–700 °C with a broad exothermic peak at 400–600 °C assigned to the fibroins’ degradation. The DTA curve of ZrMOF@SF_d_ presents a shifting of the degradation temperature around T = 360 °C (compared to the SF_d_ DTA curve at T = 320 °C), which is attributed to the combustion of the organic part of ZrMOF@SF_d_ (estimated w:w ≈ 5.7%).

The FT-IR spectra for ZrMOF and As^III^-loaded ZrMOF (Fig. 2d) are very similar, indicating the retention of the structure of the ZrMOF after As^III^ sorption. Noteworthy, in the IR spectrum of As^III^ @ ZrMOF, there is a band around 740 cm^−1^ and 1040 cm^−1^, which is attributed to As^III^-O stretch from H_3_AsO_3_^25,48,60,61^.

X-ray photoelectron (XPS) analysis was used to determine the As^III^-valence state and the eventual interaction between arsenic and the adsorbent. The high-resolution As3d XPS spectrum, shown in Fig. 2e, clearly indicates that As^III^ is the only oxidation form adsorbed onto ZrMOF sorbent. The characteristic peak at 44.2 eV corresponds to As^III^ in agreement with Sudhakar *et al*.^62^, while no peak corresponding to As^V^ is detected in As-loaded ZrMOF. This result shows that after adsorption of the As^III^ on the ZrMOF, there is no oxidation event of As^III^,  thusall the bound As atoms on ZrMOF are in the As^III ^ oxidation form. This information is in agreement with our FT-IR data, which detects the As^III^-O stretch, Fig. 2d, originating from H_3_AsO_3_. Also, the prevalence of the H_3_AsO_3_ form is corroborated hereafter by the adsorption-isotherms’ analysis, which shows that the adsorbed species is exclusively the neutral form of As^III,^ i.e., H_3_As^III^O_3_.

ZrMOF is a highly porous material with a 12-connected net based on [Zr_6_O_4_(OH)_4_] hexanuclear units interconnected via NH_2_-BDC^2−^ ligands. We should note that pZrMOF is charged due to the protonation of amine groups (as the ZrMOF is prepared in acidified water). Prior to the As^III^ sorption investigations, the ZrMOF was treated with MeOH/Et_3_N to deprotonate the  amine groups, thus resulting in a neutral framework.

**Figure 3:**
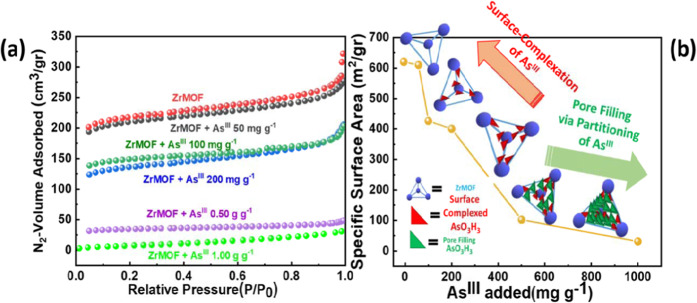
(**a**) Nitrogen sorption isotherms at 77 K for ZrMOF and As^III^@ZrMOF for different As^III^ loadings (**b**) BET surface area *vs*. As^III^ loading.

A severe decrease of the Specific Surface Area of the ZrMOF is observed upon As^III^ adsorption, see Fig. 3a,b. The non-linear [SSA *vs*. As^III^] trend in Fig. 3b, for ZrMOF can be analysed into two different domains: [i] at low As^III^-loading the SSA is decreased moderately, [ii] at high-As^III^ loading there is an abrupt lowering of the SSA. This change in SSA is not due to alteration of the crystal structure of ZrMOF, as verified by PXRD, Fig. 2b. Thus, the severe decrease of SSA upon As-uptake provides important insight into the As^III^-uptake mechanism by ZrMOF as follows: the SSA of 610 m^2^ gr^−1^ for ZrMOF is equivalent to 6.1×10^20^ nm^2^ per gram of ZrMOF. The molecular volume of H_3_AsO_3_ in H_2_O has been estimated by Canaval *et al*.^63^ to be 75 ± 10Å^3^. Accordingly, each nm^2^ surface element of ZrMOF can accommodate not more than 2 H_3_AsO_3_ molecules, which gives an N-maximum of surface-adsorbed H_3_AsO_3_ molecules N_max_ = 2 [H_3_AsO_3_ per nm^2^] × [6.1×10^20^ nm^2^ per gram of ZrMOF] ~ 1.5mmoles of H_3_AsO_3_ per gram of ZrMOF. When we compare this *vs*. the maximum As^III^-uptake capacity i.e. ~30 mmoles H_3_AsO_3_ per gram, we conclude that the experimental As-uptake is 20 times higher than the maximum As^III^-uptake capacity of 1.5 mmoles of H_3_AsO_3_ per gram, that would correspond to a mere surface coverage. Instead, the SSA drop *vs*. As-uptake data in Fig. 3b indicates a pore-filling mechanism, not a simple surface complexation. At the same time, the crystallinity of the ZrMOF material is retained after As^III^-uptake, see XRD in Fig. 2b. This result makes the ZrMOF behaving like an “As^III^-sponge” being capable of adsorbing unprecedented high-amounts, i.e., 2.2 grams of toxic As^III^ per gram of ZrMOF.

### As^III^-adsorption kinetics

**Figure 4:**
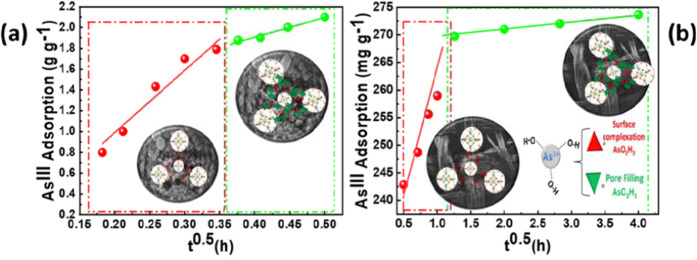
As^III^ adsorption kinetics for (**a**) ZrMOF and (**b**) ZrMOF@SF_d_ at pH 7. Symbols (, ) are experimental data. Lines are theoretical curves calculated using Eq. 2, with the parameters listed in Table S1. In (**a**) the added As^III^ concentration was 50 mg L^−1^. In (**b**) the added As^III^ concentration was 15 mg L^−1^.

Kinetic data of As^III^ adsorption by ZrMOF, Fig. 4a, show fast kinetics with a non-linear time-profile. The kinetic data can be fitted by the Weber and Morris model, described by Eq. 2^64^.

2 $$q(t)={K}_{in}{t}^{0.5}+C$$

The Weber and Morris model^64^ is based on the key-assumption that diffusivity and mass- transfer phenomena are determining the adsorption process. In Eq. 2, the fittable parameters are the kinetic constant rate *K*_*i*n_ (g g^−1^ h^1/2^), and C (g g^−1^) which is a constant depending on the type of the boundary layer^64^. Accordingly, the data in Fig. 4a can be fitted by considering two different sets of *K*_*i*n_ and C, listed in Table S1 of the Supporting Information. At early adsorption times, (red circles in Fig. 4a), a fast kinetic constant *K*_in_ = 6 g g^−1^ h^1/2^ is obtained, with C = 0.11 g g^−1^ while at prolonged adsorption times, the kinetic constant is much lower *K*_in_ = 1.9 g g^−1^ h^1/2^, with C = 1.1 g g^−1^. This analysis reveals a two kinetic-phase phenomenon for As^III^ uptake by ZrMOF. Taking into account the analysis of SSA data, we consider that the initial fast phase, corresponding to low-As uptake, is responsible for low decrease of SSA. At prolonged interaction times, where the adsorbed As^III^ is high, a slower kinetic phase is operating, which corresponds to the sharp drop of SSA, i.e. the pores of ZrMOF are filled up with H_3_AsO_3_.

**Figure 5:**
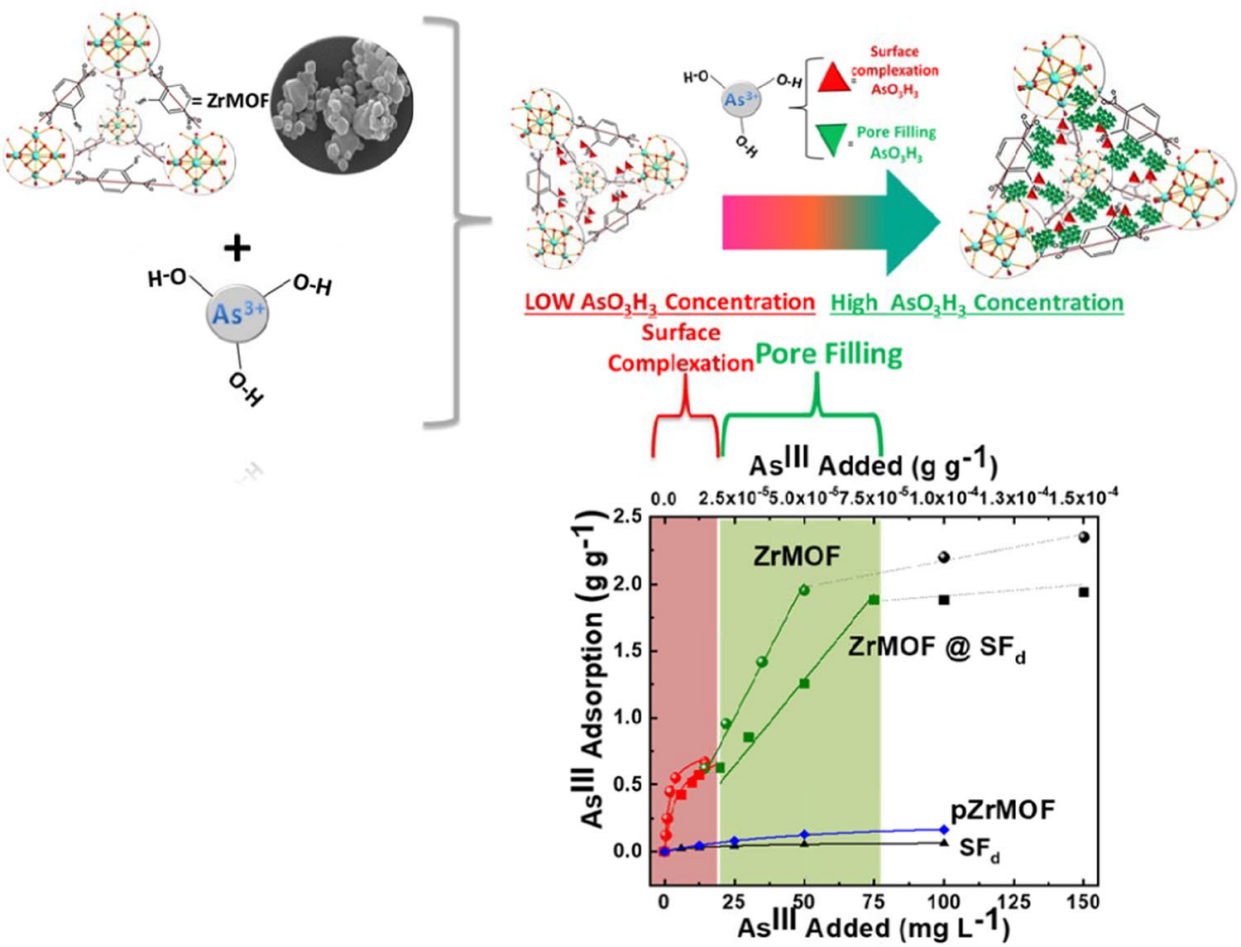
As^III^ adsorption isotherms for ZrMOF (, , •), ZrMOF@SF_d_ (, , ■),pZrMOF(), and SF_d_ (▲) at pH 7.

The same kinetic two-phase profile is observed for the ZrMOF@ SF_d_ hybrid, Fig. 4b, indicating that the grafted ZrMOF particles operate similarly, i.e. surface adsorption of As^III^ at low concentrations (red symbols in Fig. 4b) followed by pore filling at high As^III^-concentrations (green symbols in Fig. 4b). The silk fiber plays a minor role in As-uptake i.e. see adsorption isotherm in Fig. 5.

### As^III^-adsorption isotherms

A non-linear isotherm characterizes the uptake of As^III^ by ZrMOF, see Fig. 5(, ), which can be analyzed in the two regions depending on the initial concentration of As^III^. [i] At low As^III^-concentrations (<25 mg As^III^ Lt ^−1^), the isotherm shows a Langmuir-like trend, see solid-red symbols in Fig. 5(). [ii] At increased initial As^III^ concentrations, the isotherm data show a linear As-uptake isotherm, Fig. 5(). This trend continues up to 75–80 mg of added As^III^ Lt ^−1^. At even higher initial As^III^, the isotherm flattens, indicating a saturation of the As^III^-uptake by ZrMOF. Based on the data of Fig. 5, the maximum adsorbed As^III^per gram of ZrMOF material corresponds to a maximum of 2200 mg As^III^ gr^−1^ of ZrMOF. The ZrMOF@SF_d_ material exhibited a similar two-isotherms profile, see Fig. 5(, ) When normalized [per mass of grafted ZrMOF], the As^III^ -uptake data in Fig. 5(, ) show that the performance of the ZrMOF@SF_d_ material is within ~10% comparable to ZrMOF. Thus, grafting of ZrMOF retains its As^III^ -uptaking capacity. For reference, pZrMOF and the SF_d_ alone Fig. 5(), (▲) show a very low As^III^ -uptake, i.e. 0.260 g g^−1^, and 0.068 g g^−1^ respectevelly. The significant inhibitory effect of the cation sites in cationic pZrMOF, to As^III^ uptake i.e. *vs* the neutral ZrMOF, reveal that the surficial NH_2_ sites play key role in the uptake mechanism. This is further analyzed in the following in the theoretical surface Complexation Modeling hereafter.

Theoretical modeling of the data in Fig. 5 can be done using two isotherm-adsorption models. [a] *At low added-As*^*III*^ *concentrations*, a Langmuir equation (Eq. 3) describes adequately the process, see fit (red line in Fig. 5) where q_m_ (mg g^−1^) is maximum As^III^ adsorption, q^L^_ADS_ (mg g^−1^) is the surface concentration of adsorbed As^III^ species in materials. C_e_ (mg L^−1^) is the initial As-concentration. K_Langmuir_ is the Langmuir stability constant representing the strength of As^III^-binding of the ZrMOF surface^65^.

3 $${q}_{{\rm{ADS}}}^{L}=\frac{{{q}_{m}}^{\ast }{{K}_{{\rm{Langmuir}}}}^{\ast }{C}_{e}}{1+{{K}_{{\rm{Langmuir}}}}^{\ast }{C}_{e}}$$

[b] *At increased added-As*^*III*^ *concentrations*, we consider a linear Freundlich-type isotherm (Eq. 4)

4 $${q}_{{\rm{Ads}}\,}^{{\rm{Part}}}={K}_{{\rm{part}}}{C}_{e}^{\frac{1}{n}}$$

where q^Part^ gives the bound As^III^ -moieties in the ZrMOF, in mg g^−1^. The index n reflects a constant related to the intensity of sorption or the degree of the dependence of sorption on concentration. The efficiency of uptake is classified according to the value of K_part_. The linear Freundlich-type adsorption isotherms can be used to describe pore filling/partitioning processes^66^ in hydrophobic/hydrophilic interfaces.

Using the two isotherms Eqs. 3 and 4, the data in Fig. 5 can be fitted (see solid lines in Fig. 5) with the parameters listed in Table 1. More specifically, at low As^III^-concentrations, the uptake capacity -due to surface complexion- can achieve a maximum of q_m_ = 0.83 g g^−1^of As^III^ at pH = 7. At high As^III^-concentration, where pore filling is operating, Fig. 5 (green line) a maximum As-uptake is attained of q_e_ = 2.2 g g^−1^of As^III^ at pH = 7.

**Table 1:** Parameters for Langmuir isotherms and Freundlich isotherms, used to fit the experimental data for As^III^ binding onto pZrMOF, ZrMOF, SF_d,_ and ZrMOF*@ SF*_*d*_ at pH 7.

Materials	Langmuir			Freundlich			
	q_m_(g g^−1^)	K_ads_	R^2^	q^Part^(g g−1)	n	K_Part_	R^2^
*ZrMOF*	0.83	0.18	0.990	2.20	1	0.04	0.989
*ZrMOF@ SF*_*d*_	0.14	0.063	0.988	1.80	1	0.0045	0.974
*pZrMOF*	0.017	0.260	0.994				
*SF*_*d*_	0.071	0.068	0.984				

Further analysis of the surface adsorption for the As^III^ species can be done by modeling of the pH-dependent As-uptake on the ZrMOF. This analysis, detailed in our previous works^16,24,67^, is described in Supporting Information, Fig. S4. According to Fig. S4, the pH-dependent profile for low As^III^-concentrations shows that As^III^ binds in its neutral form H_3_AsO_3_ at the neutral amino-sites of neutral ZrMOF. This result is in agreement with Georgiou *et al*.^16^, Gupta *et al*.^27^, Su and Puls^28^. The surface amines ≡NH_2_ act a specific binding sites for As^III^-uptake, see reaction (15), and (17) ≡NH_2_ + H_3_AsO_3_ ↔ ≡NH_2_-[H_3_AsO_3_] in Table S2. We underline that in the cationic pZrMOF, the protonated ≡NH_3_^+^ sites, reaction (12) in Table S2, do not favor adsorption of As^III^ and this is the origin of the inferior performance of pZrMOF *vs*. ZrMOF. This is structurally described in Fig. S4d.

Since, in natural waters, several ions may coexist with arsenic, these can potentially compete with As-uptake^68^. In this context, the impact of competing ions such as PO_4_^3−^, CO^2−^_3,_ NO_3_^−^, ^SO2−^_4_, Cl^−^ and HCO_3_^−^ on As^III^ adsorption was studied, see Fig. S5 in the Supporting Information. The data in Fig. S5, indicate that ZrMOF and ZrMOF@ SF_d_ effectively remove As^III^ even in the presence of CO_3,_ NO_3_^−^, SO_4_^2–^, Cl^−^ and HCO_3_^−^. The stronger inhibitory effect is exerted by PO_4_^3−^ ions which may inhibit As^III^ adsorption by 40% and 60% for ZrMOF and ZrMOF@SF_d,_ respectively. The results are well agreement with Sudhakar^62^ *et al*. and Jain and Loeppert^69^, which point out that the natural water ions do not affect the As^III^ adsorption except for PO_4_^3^ which destabilizes the MOF structure.

Finally, we have evaluated the possibility of reusing the ZrMOF and ZrMOF@SF_d_ materials after regeneration. Thus, we have applied the regeneration protocol^24,70^, which involved high ionic-strength treatment. More particularly, the protocol involves incubation for 24 hours under stirring at a pure aqueous solution containing 1 M of KNO_3_. Our data show that the As^III^ adsorbed on either ZrMOF or ZrMOF@SF_d_ cannot be removed by this treatment, indicating the high stability of the bound As^III^, i.e. attributed to its irreversible penetration into the pores of the ZrMOF.

### Comparison of As^III^-uptake with similar metal-organic framework materials

**Figure 6:**
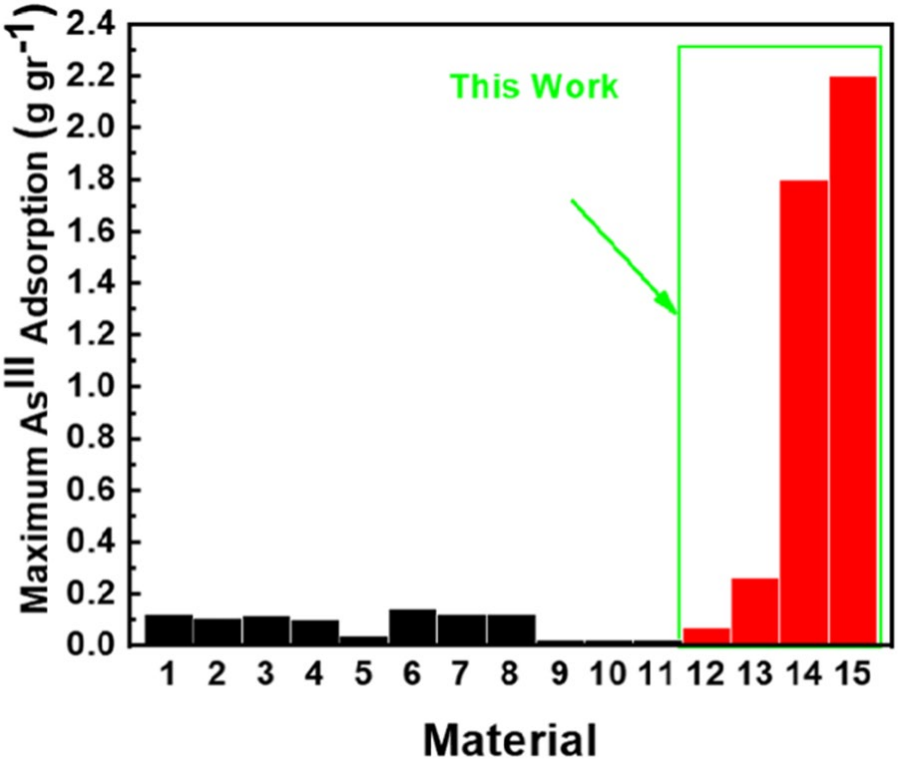
Maximum As^III^ adsorption capacity (g g^−1^) at waters’ near-neutral pH of some adsorbents reported in the literature compared with the present materials: 1) ZIF-8(cubic)^77^, 2) ZIF-8(leaf)^77^, 3) ZIF-8 (dodecahedral)^77^, 4) Fe_3_O_4_@ZIF-8^78^,5) HCl-UiO-66(SH)_2_^79^, 6) CoFe_2_O_4_@MIL-100(Fe)^48^, 7) Fe_3_O_4_@MIL-101^80^, 8) MIL-100(Fe)^16^, 9) M600^16^, 10) M800^16^, 11) M900^16^, 12) ZrMOF(this work), 13) cationic pZrMOF (this work), 14) SF_d_ (this work), 15)neutral ZrMOF@SF_d_ (this work).

Figure 6 summarizes a comparison of As^III^ sorption by the present materials *vs*. other pertinent MOF-based materials reported in the literature. According to Fig. 6, ZrMOF supersedes by far any known material.

This result is attributed to the fundamentally different mode of action of the neutral ZrMOF, i.e. the partitioning-like mechanism resulting in pore-filling allows exploitation of the full pore volume as a “sponge” for the uptake of the As^III^ species form solution.

## Conclusions

Using XPS, FTIR, BET-porosimetry data, with theoretical Surface-Complexation-Modeling (SCM), we report a two-step  phenomen non  which boosts high-As^ΙΙΙ^-uptake. First, at low As^III^-concentrations, surface-complexation of H_3_AsO_3_ results in As^III^-coated voids of neutral ZrMOF, and subsequently, at high As^III^-concentrations, the As^III^-coated voids of neutral ZrMOF are filled-up by H_3_AsO_3_ *via* a partitioning-like mechanism. Also, we present an innovative hybrid-material, ZrMO*F@SF*_*d*_ operating like an “As^III^-sponge” with unprecedented efficiency of 1800 mg As^III^ gr^−1^. ZrMOF@*SF*_*d*_ consists of a Zirconium Metal-Organic Framework [ZrMOF] covalently grafted on *SF*_*d*_. ZrMOF itself exhibits As^III^ adsorption of 2200 mg gr^−1^, which supersedes any -so far- known As^ΙΙΙ^-sorbent. The reference materials i.e. cationic-pZrMOF and SF_d_ play secondary role in As^III^-adsorption with adsorption capacity 260 mg As^III^ gr^−1^ and 68 mg As^III^ gr^−1^ respectively. Finally, the present research exemplifies for the first time a novel concept of a “partitioning-like” mechanism, operating for adsorption of H_3_AsO_3_ , by neutral metal oxide materials. So far, such a mechanism has been conceptualized only for the uptake of non-polar organics by natural organic matter or synthetic polymers.

## Methods

### Materials

Sodium meta-arsenite NaAsO_2_ was obtained from Sigma-Aldrich, while HCl, NaOH, KNO_3,_ and Cu (NO_3_) ∙ 3H_2_O obtained from Merck. 2-(N-Morpholino)ethanesulfonic acid hydrate, 4-Morpholineethanesulfonic acid (call MES hydrate) & 4-(2-Hydroxyethyl)piperazine-1-ethanesulfonic acid, N-(2-Hydroxyethyl)piperazine-N-(2-ethanesulfonic acid)(call HEPES), used for pH buffering, were obtained from Sigma-Aldrich. Milli-Q Academic system, Millipore produced ultrapure water.

The Silk Fabric (SF) provided by Tsiakiris Georgios Silk Company, Alexandroupoli, Greece. Sodium carbonate (Na_2_CO_3_) purchased from Riedel de Haën. The coupling agent 3-(chloropropyl)trimethoxysilane was provided by Fluka. Methanol and ethanol purchased from Merck and diethyl ether from Sigma Aldrich.

All reagents were of analytical reagent grade purity, and all solutions prepared using deionized water obtained with a Milli-Q system with a conductivity of 18.2 μS cm^−1^.

### ZrMOF preparation

The protonated (cationic) [Zr_6_O_4_(OH)_4_(NH_3_^+^-BDC)_6_]Cl_6_ ∙ 35H_2_O (herein codenamed as pZrMOF) was synthesized as described previously^71^. To prepare the neutral material, pZrMOF (100 mg, 0.038 mmol) was treated with Et_3_N (72.6 mg, 0.7 mmol) in MeOH (4 mL) for 1 h. The resulting solid [Zr_6_O_4_(OH)_4_(NH_2_-BDC)_6_]∙xMeOH∙yH_2_O (herein codenamed as ZrMOF) was then isolated by filtration, washed with MeOH and dried in the air. Yield: 89%.

### Degumming process of Silk Fabric (SF_d_)

The SF cut in pieces of 3.3 × 0.9 cm (≈15 mg), which were immersed into a 200 ml round-bottom flask and were degummed in a 0.05 wt.% Na_2_CO_3_/H_2_O solution at 90 °C for 30 min and then rinsed thoroughly with double distilled water to extract the sericin protein and other impurities. This process repeated three times to obtain pure silk fibroin fibers (SF_d_). The degummed silk fibroin fibers (SF_d_) dried at 40 °C under atmospheric pressure.

### Covalent grafting of ZrMOF on SF_d_ (ZrMOF@SF_d_)

A solution of ZrMOF (0.066 mmol) in 10 ml of methanol prepared for sonicating to achieve a good suspension in the dispersion media. After 0.022 mmol of 3-(chloropropyl) trimethoxysilane sonicated added in the solution, and finally, the reaction mixture refluxed at 60 °C for 48 h. The molar ratio of ZrMOF/silane was 3:1. To this, 30 mg of degummed SF_d_ fibers and 5 ml of ethanol were added and refluxed at 60 °C for 24 h. The degummed SF_d_ fibers (30 mg) immersed into 10 ml of ethanol for 24 h, before modification with ZrMOF/silane. After cooling at room temperature, the resulting material, ZrMOF@SF_d_ was washed several times with methanol, ethanol, and diethyl ether and dried under vacuum at 40 °C for 24 h.

### Physical characterization of materials

EDS analysis for ZrMOF showed no Cl confirming the complete deprotonation of ammonium groups. The powder X-ray diffraction (PXRD) measured at room temperature on an STOE-STADIMP powder diffractometer. PXRD equipped with an asymmetrically curved Germanium monochromator (CuKα1 radiation, λ = 1.54056 Å) and a one-dimensional silicon strip detector (MYTHEN2 1 K from DECTRIS). The line focused Cu X-ray tube operated at 40 kV and 40 mA. Powder of each sample was packed in a 1 mm diameter polyimide capillary (polymer substrate with neither Bragg reflections nor broad peaks above 10 °) and measured in Debye-Scherrer geometry on a spinning stage (~200 rpm). Intensity data from 3 to 125 degrees 2Θ collected for 17 h with a step of 0.005 degrees. The instrument calibrated against a NIST Silicon standard (640d) before the measurement.FT-IR spectra were recorded on KBr pellets in the 4000-400 cm^−1^ range using a Perkin-Elmer Spectrum GX spectrometer. Thermogravimetric analyses (TGA) were performed on a NETZSCH STA 449 C system. Sample analysis was conducted from 25 to 900 °C in an air atmosphere (50 mL min^−1^ flow rate) with a heating rate of 10 °C min^−1^. Scanning electron microscope (SEM) performed by a JEOL JSM-6390LV equipped with an Oxford INCA PentaFET-x3 energy-dispersive X-ray spectroscopy (EDS) detector. Data acquisition performed with an accelerating voltage of 20 kV and 120 s accumulation time. Then the images were taken with a field emission JEOL JSM 7000 F electron microscope operating at 15 kV accelerated voltage. The samples were sputter-coated with 5–10 nm also reduce charging by used Au film.

*X-Ray Photoelectron Spectroscopy (XPS)* measurements were using a SPECS GmbH. Instrument equipped with a monochromatic MgKα source (*h*ν = 1253.6 eV) and a Phoibos-100 hemispherical analyzer. The spectra were recorded under ultra-high vacuum s with a base pressure of 2–5 × 10^–10^ mbar. Prior to measurement, the samples were placed on silicon substrates under high vacuum, before being placed in the main chamber for XPS measurement. The take-off angle was 45°. The recorded spectra were the average of three scans, with energy step 0.1–0.2 eV and a dwell time of 1 s. The As3d binding energy is calibrated based on the C1s core level at 284.5 eV. The spectral analysis included [i] a Shirley background subtraction, and [ii] peak deconvolution employing mixed Gaussian–Lorentzian functions in a least-squares curve-fitting program (WinSpec, Laboratoire Interdisciplinaire de Spectroscopie Electronique, University of Namur, Belgium)^72,73^.

N_2_ adsorption isotherms were measured at 77 K using a Quantachrome NOVAtouch LX^2^. Before analysis, all samples were degassed at 80 °C under vacuum (<10^–5^Torr) for 16 h. The specific surface areas were calculated by the Brumauer-Emmett-Teller (BET) method using the N_2_-adsorption data points, in the relative-pressure range P/P_o_ of 0.05–0.35.

### As^III^ analytical determination

The concentration of As^III^ in the aqueous solution determined by square wave Cathodic Stripping Voltammetry (SW-CSV) using a Trace Master5-MD150 polarograph by Radiometer Analytica. SW-CSV is well suited for the analytical determination of As^III^ ^22,74^ with a low detection limit (0.5 μg L^−1^). The measuring borosilicate glass cells obtained from Radiometer Analytica. The working electrode was a hanging mercury drop electrode (HMDE) with drop diameter of 0.4 mm generated by a 70 μm capillary. An Ag/AgCl electrode with a double liquid junction used as the reference electrode with a Pt measuring electrode. Importantly, samples were not purged with N_2_ gas to avoid the loss of As^III^. During the stripping step, the solution stirred at 525 rpm. All measurements we performed using aliquots of 8.3 mL shifting at pH < 0.5 by 1.5 mL from 6.66 M of HCl and final 2 M concentration in the electrochemical cell, then 8 ppm of Cu^2+^ were added. In the following, As^III^ was determined by SW-CSV with accumulation potential E = −400 mV and accumulation time in the 60 s. As^III^ quantified by its signal at E_1/2_ = −670 mV^16,19,22^.

### As^III^ adsorption experiments

*For* the kinetic measurements, As^III^ uptake from aqueous solutions studied in batch experiments. The kinetics of As^III^ adsorption using ZrMOF studied as follows: 4 10^–4^ g L^−1^ of *ZrMOF* were dispersed in 50 mL buffered aqueous solution in polypropylene tubes at pH 7, in the presence of 50 mg L^−1^ As^III^. For samples, ZrMOF @ SF_d_ 3.4 mg was dispersed in 25 mL buffered aqueous solution in polypropylene tubes at pH 7, in the presence of 15 mg L^−1^ As^III^. The time-evolution of As^III^ concentration was monitored at contact times ranging between 0–240 min and 0–960 min, respectively. At the end of each contract period, all sample centrifugation and the supernatant solution analyzed for As^III^. To determine the adsorption rates of As^III^, the amount of As^III^ adsorbed at time t, q (mg As^III^ g^−1^), calculated from the mass-balance between the initial concentration and the concentration at time t onto the solid adsorbents^16,29^.

Adsorption isotherms for pZrMOF and ZrMOF were recorded at pH 7 in the presence of 0–100 mg L^−1^ NaAsO_2_ interacting with 0.1 g L^−1^ pZrMOF and 0 to 150 mg L^−1^ NaAsO_2_, interacting with 4 10^–4^ g L^−1^ of ZrMOF suspended in 50 mL buffer solution in polypropylene tubes. On the other hand, for *SFd and* ZrMOF @ SF_d_, 0 to 100 mg L^−1^ and 0 to 150 mg L^−1^ NaAsO_2_ and 0.2 g L^−1^,0.14 g L^−1^ respectively were suspended in 25 mL buffer solution in polypropylene tubes.

pH-dependent (pH-edge) experiments allow a detailed probing of the interfacial adsorption *mechanisms*^16,29^, while adsorption isotherms report the maximum uptake capacity. In this work, pH-edge adsorption experiments carried out for an initial concentration of 5, 5, and 15 mg L^−1^(NaAsO_2_) and also 0.1 g L^−1^,0.4 10^–4^ g L^−1^, 0.14 g L^−1^ of pZrMOF, *ZrMOF and ZrMOF @ SF*_*d*_ respectively, suspended in 50 mL buffer solution whose pH adjusted in the range 4 to 8, in polypropylene tubes.

After metal addition, the suspension was allowed to equilibrate for 15 min ZrMOF, *and pZrMOF* at RT, while agitated using a magnetic stirrer. After completion of equilibration, the suspensions centrifuged at 6000 rpm for 10 min, and the supernatant solutions were analyzed for As^III^ as described above. For *SF*_*d*_ and ZrMOF *@ SF*_*d*_ after metal addition, the suspension was allowed to equilibrate overnight at RT while using a magnetic stirrer. After completion of equilibration, the *SF*_*d*_ or ZrMOF *@ SF*_*d*_ suspension collects by metal tweezer.

Reuse experiments were also conducted for ZrMOF, which had adsorbed As^III^ at pH 7. To reuse the samples, we had to desorb the adsorbed As. Thus, following the method used in ref. ^30,75^, after As^III^ adsorption, the material was immersed in an aqueous solution of 1 M KNO_3_ for 24 h, and the supernatant was analyzed for As^III^ release^24,70,76^. Similarly, the ZrMOF @ SF_d_ once loaded with As^III^ were washed at high ionic strength 1 M KNO_3_ and the supernatant was analyzed for As^III^ release.

Control experiments (without ZrMOF, pZrMOF, SF_d_, and ZrMOF @ SF_d_) showed no loss of initial As^III^. The initial pH values of buffer solutions were adjusted to the requested using small volumes of 1 M HCl or 1 M NaOH. It should mention that HCl is inert towards As^III^ in voltammetric measurements, the pH drift of each suspension, i.e. measured at the beginning and the end of incubation, was <0.2 pH units.

## Supplementary information

Supplementary information.
