# Supplementary material for: A Hybrid {Silk@Zirconium MOF} Material as Highly Efficient AsIII-sponge
Source: Sci Rep. 2020 Jun 9;10:9358. doi: 10.1038/s41598-020-66091-w (PMC7283345; doi:10.1038/s41598-020-66091-w)
Supplement: Supplementary file 1 — Supplementary information. [file 41598_2020_66091_MOESM1_ESM.docx]

**A Hybrid {Silk@Zirconium MOF} Material as Highly Efficient As^III^-sponge**

Yiannis Georgiou^1,2^, Sofia Rapti^1^, Alexandra Mavrogiorgou^1^, Gerasimos Armatas^3^, Manolis J. Manos^1, 1a^, Maria Louloudi^1,1a^, Yiannis Deligiannakis^2,1a,1b^ *

^1^Laboratory of Biomimetic Catalysis and Hybrid Materials, Department of Chemistry, University of Ioannina, GR45110, Greece

^2^Laboratory of Physical Chemistry of Materials and Environment, Department of Physics, University of Ioannina, GR45110, Greece

^3^Department of Materials Science and Technology, University of Crete, Heraklion 71003, Greece

1^a^ Institute of Materials Science and Computing, University Research Center of Ioannina, GR45110, Greece

^1b^ Institute of Environment & Sustainable Development, University Research Center of Ioannina, GR45110, Greece


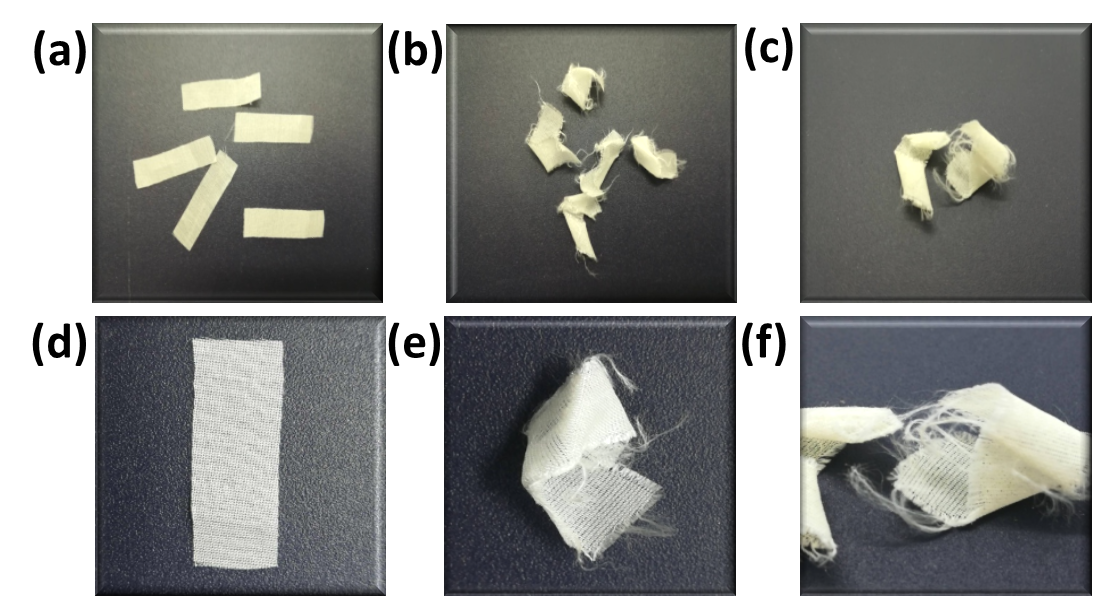


***Scheme S1: (a)*** *Silk fabric (SF)* ***(b)*** *Silk fabric* ***(SF_d_)*** *after degumming process and* ***(c),(d),(e),(f)*** *Silk fabric* ZrMOF@**SF_d_** *after covalent grafting of Zr-MOF.*

*
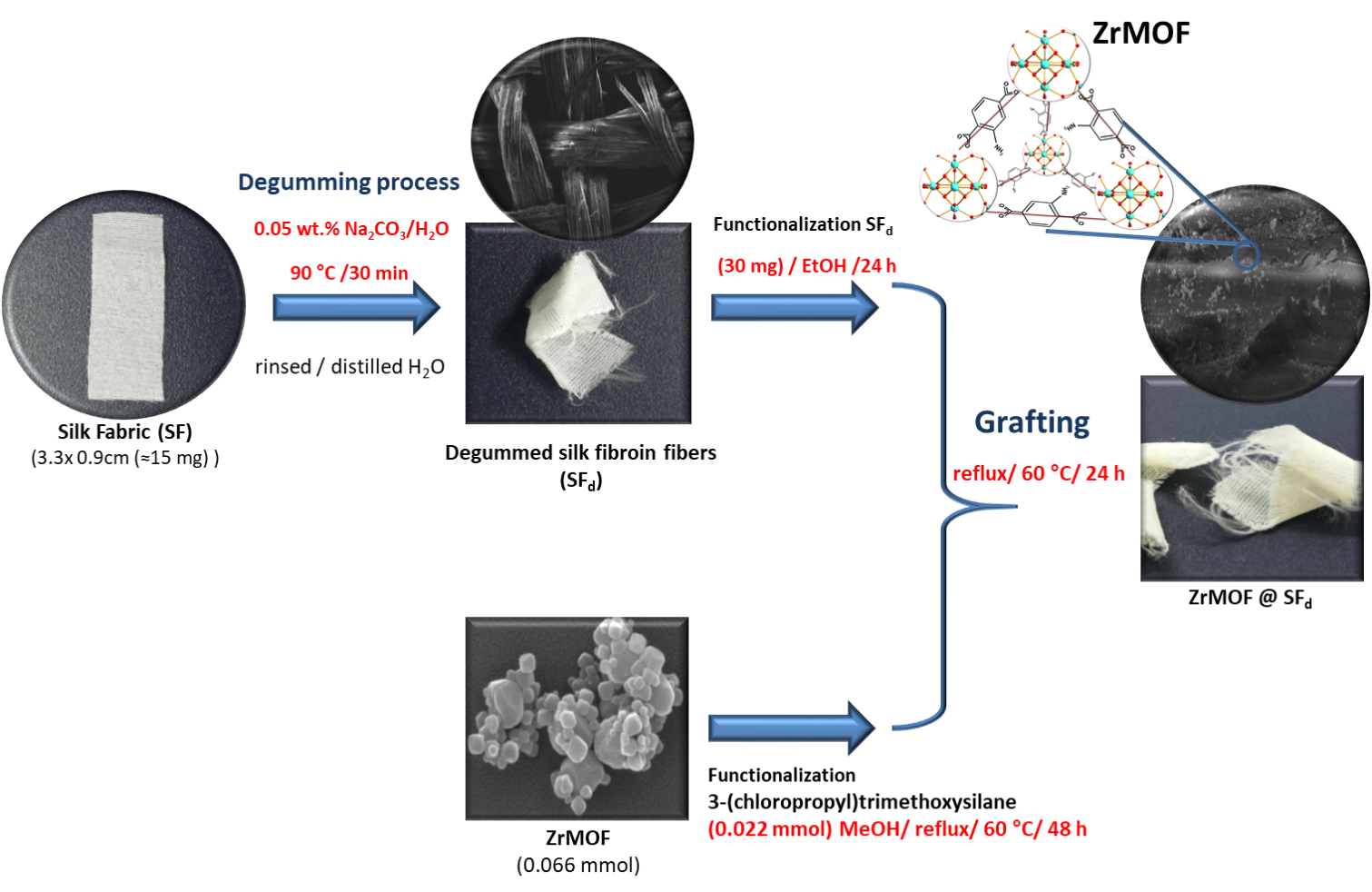
*

***Scheme S2:*** *Description of the grafting process of ZrMOF of the degummed silk SF_d_.*

**Degumming Processes:** Silk Fabric **(SF)** degummed in a 0.05 wt.% Na_2_CO_3_/H_2_O solution at 90 °C for 30 min (repeated three times to obtain the pure degummed silk fibroin fibers **(SF_d_)**) **.** Finally SF_d_ was dried at 40 °C under atmospheric pressure.

**Covalent grafting of ZrMOF on SF_d_ fibers:** solution of **ZrMOF** (0.066 mmol) and 3-(chloropropyl)trimethoxysilane (0.022 mmol) refluxed at 60 °C for 48 h(ZrMOF /silane was 3:1). Then SF_d_ (30 mg) immersed into 10 ml of ethanol for 24 h, to modification with ZrMOF /silane. After cooling at room temperature, the resulting material **ZrMOF @ SF_d_** was washed several times with methanol, ethanol and diethylether and dried under vacuum at 40 °C for 24 h.


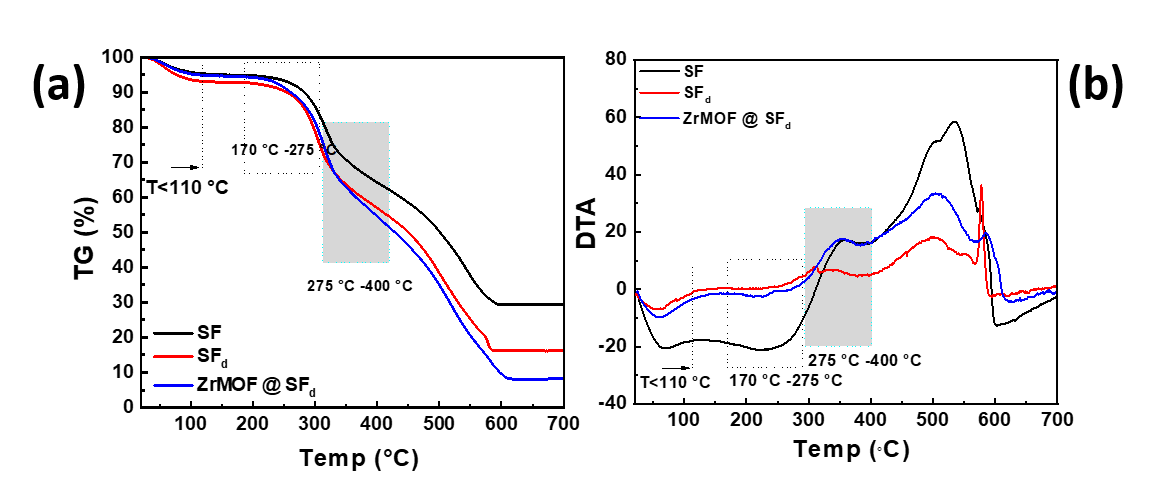


**Figure S1** :(a)TGA (solid lines) and (b) first derivative (DTG) (solid lines) plots for SF, SF_d_ and ZrMOF@ SF_d_

The analysis of the thermogravimetric analysis data, has been done according to: Annie Xi Lu et.al. ACS Appl. Mater. Interfaces 2017, 9, 15, 13632-13636 ([doi /10.1021/acsami.7b01621](https://doi.org/10.1021/acsami.7b01621)) as follows.

[1] Estimation of Mass of ZrMOF grafted on the in ZrMOF@SF_d_ hybrid

Mass of ZrMOF @ SF_d_ (0.17 mg after TGA ) at 700 ℃ * $\frac{mass of ZrMOF at 375 ℃(8,12 mg)}{mass of ZrMOF at 700 ℃(5.86 mg)}=$

=>0.17 mg (700 ℃)*$\frac{8,12 mg(375 ℃)}{5.86 mg(700 ℃)}$ = 0.235 mg Mass of ZrMOF in ZrMOF @ SF_d_

[2] Estimation % ZrMOF in ZrMOF @ SF_d_

Mass of ZrMOF @ SF_d_ at 375 ℃ 🡪 4.07 mg

$\frac{0.235 mg}{4.07 mg(375℃)}*100=0.0577*100=5.7 \%$

**Figure S2:** FT-IR spectra for SF, SF_d_ and ZrMOF@ SF_d_

**Figure S3:** pXRD for Silk, SF_d_ and ZrMOF @ SF_d_ respectively

|  | **Table S1** The constants for the Kinetics model for AsIII binding onto ZrMOF and ZrMOF *@ SF_d_* and at pH 7. | | |
| --- | --- | --- | --- |
|  |  | **15 mg L^-1^** | |
|  | **Materials** | **K_in_(g g^-1^ h^1/2^)** | **C(g g-1)** |
|  | ZrMOF*@ SF_d_(green cube)* | 0.0013 | 0.0026 |
|  | ZrMOF*@ SF_d_(red cube)* | 0.046 | 0.0021 |
|  |  | **50 mg L^-1^** | |
|  | ZrMOF(green sphere) | 6 | 0.11 |
|  | ZrMOF(red sphere) | 1.9 | 1.1 |
|  |  |  |  |
|  |  |  |  |

Surface complexation *Modeling (SCM)*: The pH-edge data modeled using SCM ^1–3^ that assumes the interfacial and solution reactions only at surface As^III^ adsorption and detailed in Table S1. In the fit, we have used a Diffuse Layer model (Table S2) with a capacitance C=22 μF cm^-1^.

In Table S2, the symbol “≡” stands for surface species of the ZrMOF, and ZrMOF @ SF_d_. Among all reactions in Table S2, the key-one for As^III^ uptake are reactions (9),(11) ≡ZrΟH + H_3_AsO_3_  ≡Zr-[HAsO_3_] + H_2_O .

Theoretical fit entails that As^III^ binds in its neutral form H_3_AsO_3_ with the neutral Zr-sites ≡ZrOH. This result is in agreement with Georgiou et al. .^1^, Gupta et al. ^4^, Su, and Puls^5^. Furthermore, the surface amines ≡NH_2_ contribute a higher As^III^-uptake, reaction (10), and (12) ≡NH_2_ + H_3_AsO_3_  ≡NH_2_-[H_3_AsO_3_] in Table S1.

The stability constants derived by the fit to the data, see red and blue symbols in Figure 6, are The stability constants derived by the fit to the data, see red and green symbols in Figure 6, are log*K*_[≡ZrOH -[ H3AsO3 ]]_=3.8, log*K*_[≡NH2-[ H3AsO3 ]]_ =2.1 and log*K*_[≡NH2-[ H3AsO3 ]]_ =3 listed in Table S2. These two reactions provide the main reaction as that describes the experimental pH-profile successfully.


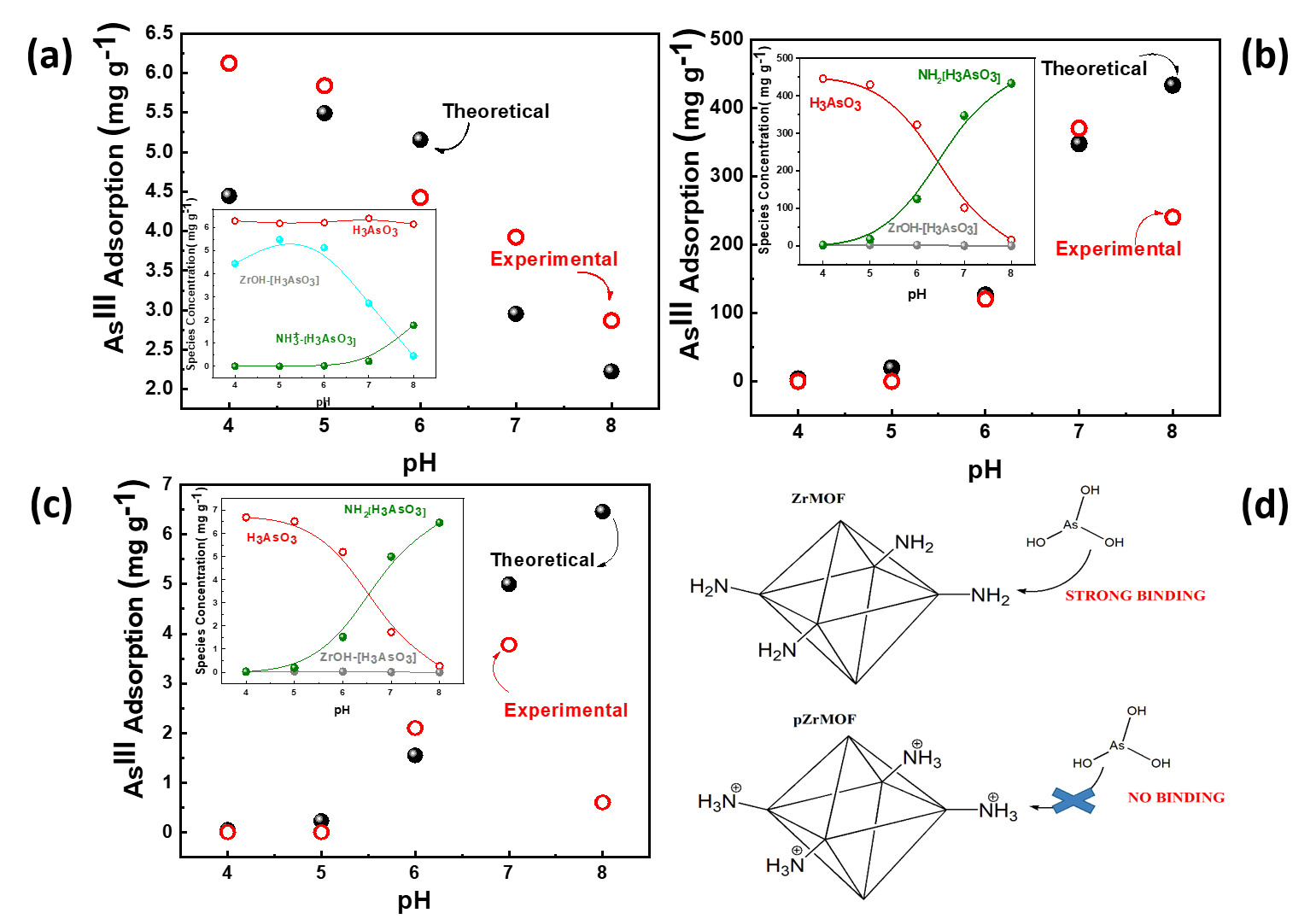


**Figure S4** As^III^ adsorption in the pH range 4 to 8 for (a)ZrMOF (b) pZrMOF, and (c)ZrMOF @ SF_d_: Import figure pH range 4 to 8 for As^III^ adsorption onto ZrMOF and ZrMOF @ SF_d_.(d) Effect of As^III^-adsorption onto neutral ZrMOF and cationic pZrMOF.

The ensuing speciation, see Figure S4 for the ZrMOF and ZrMOF @ SF_d_, shows that we notice that for both the strong log*K*_[≡NH3 -[ H3AsO3 ]]_ =3.8, 2.1 and 3.6, i.e., the strong affinity of the neutral ≡sites, is responsible for the As^III^ uptake in both materials. The contribution of the amine groups, despite their non-negligible log*K*_[≡NH2-[ ZrOH ]],_ is minor. The theoretical analysis in Figure S4 shows that neutral ZrMOF has a strong affinity for As^III^ adsorption. The ≡NH_2_ sites in ZrMOF are strong As^III^ uptake sites.

| **Table S2** Equilibrium equations and optimized constants of reactions for As^III^ binding onto pZrMOF,ZrMOF, and ZrMOF @SF_d_ | | |
| --- | --- | --- |
| **Reaction** | **Log *K*** | **References** |
| ***Solution reactions*** |  |  |
| ***Protonation of* As^III^** |  |  |
| 1) H_3_AsO_3_↔ H^+^+ Η_2_AsO^-^_3_ | -9.2 ± 0. 2 | ^1,3,6^ |
| 2) H_2_AsO^-^_3_↔ H^+^+ ΗAsO_3_^2-^ | -21.2 ± 0.2 | ^1,3,6^ |
| ***Protonation of* pZrMOF** |  |  |
| 3) ≡ZrOH + H^+^ ↔≡ZrΟH_2_ | -3.5 ± 0. 2 | This work |
| 4) ≡ZrOH ↔≡ZrO^-^ + H^+^ | -6.9 ± 0.2 | This work |
| **Protonation of ZrMOF** |  |  |
| 5) ≡ZrOH + H^+^ ↔≡ZrΟH_2_ | -3.5 ± 0. 2 | This work |
| 6) ≡ZrOH ↔≡ZrO^-^ + H^+^ | -6.9 ± 0.2 | This work |
| 7)≡NH_2_ + H^+^ ↔ ≡NH_3_^+^ | 8.9 ± 0.2 | This work |
| **Protonation of ZrMOF @ SF_d_** |  |  |
| 8) ≡ZrOH + H^+^ ↔≡ZrΟH_2_ | -3.5 ± 0. 2 | This work |
| 9) ≡ZrOH ↔≡ZrO^-^ + H^+^ | -6.9 ± 0.2 | This work |
| 10)≡NH_2_ + H^+^ ↔ ≡NH_3_^+^ | 8.9 ± 0.2 | This work |
| ***Surface reactions*** |  |  |
| ***Sorption of As^III^ onto pZrMOF*** |  |  |
| 11) ≡ZrΟH + H_3_AsO_3_ ↔ ≡Zr -[ HAsO_3_ ] + H_2_O | 12.9 ± 0.2 | This work |
| 12) ≡NH_3_ + H_3_AsO_3_ ↔ ≡NH_3_-[H_3_AsO_3_] | 1 ± 0.2 | This work |
| ***Sorption of As^III^ onto* ZrMOF** |  |  |
| 13) ≡ZrΟH + H_3_AsO_3_ ↔ ≡Zr -[ HAsO_3_ ] + H_2_O | 2 ± 0.2 | This work |
| 14) ≡NH_2_ + H_3_AsO_3_ ↔ ≡NH_2_-[H_3_AsO_3_] | 2.1 ± 0.2 | This work |
| ***Sorption of As^III^ onto* ZrMOF @ SF_d_** |  |  |
| 15) ≡ZrΟH + H_3_AsO_3_ ↔ ≡Zr-[ HAsO_3_ ] + H_2_O | 0.2 ± 0.02 | This work |
| 16) ≡NH_2_ + H_3_AsO_3_ ↔ ≡NH_2_-[H_3_AsO_3_] | 3.6 ± 0.1 | This work |
| ***Diffuse layer model (25 ºC)***  *Concentration of suspended solid:* 0.1 g L^-1^ (pZrMOF), 0.4 10^-4^ g L^-1^ (ZrMOF), 0.14 g L^-1^ (ZrMOF@ SF_d_),  *Concentration of electrolyte: 0 mol L^-1^* (pZrMOF),*0 mol L^-1^* (ZrMOF), *0 mol L^-1^* (ZrMOF@ SF_d_)..  Constant capacitance : 22 μF cm^-1^ (pZrMOF),22 μF cm^-1^ (ZrMOF), 22 μF cm^-1^  (ZrMOF@ SF_d_) | | |

**Figure S5** Investigation of the effect of competing ions on As^III^ adsorption for the ZrMOF and ZrMOF @ SF_d_ materials concentration of 50 mg L^-1^ for As^III^ separately. 100 mL arsenic-contaminated deionised water taken with and the incubation time kept as 120 min for ZrMOF and 24h for ZrMOF @ SF_d_ . Ion concentration 1 mM, was added prior to As^III^ addition in water.

**References**

1. Georgiou, Y., Perman, J. A., Bourlinos, A. B. & Deligiannakis, Y. Highly Efficient Arsenite [As(III)] Adsorption by an [MIL-100(Fe)] Metal-Organic Framework: Structural and Mechanistic Insights. *The Journal of Physical Chemistry C* **122**, 4859–4869 (2018).

2. Georgiou, Y. *et al.* Surface decoration of amine-rich carbon nitride with iron nanoparticles for arsenite (AsIII) uptake: The evolution of the Fe-phases under ambient conditions. *Journal of hazardous materials* **312**, 243–253 (2016).

3. Petala, E. *et al.* Magnetic Carbon Nanocages: An Advanced Architecture with Surface- and Morphology-Enhanced Removal Capacity for Arsenites. *ACS Sustainable Chemistry & Engineering* **5**, 5782–5792 (2017).

4. Gupta, A., Yunus, M. & Sankararamakrishnan, N. Zerovalent iron encapsulated chitosan nanospheres–A novel adsorbent for the removal of total inorganic Arsenic from aqueous systems. *Chemosphere* **86**, 150–155 (2012).

5. Su, C. & Puls, R. W. Arsenate and Arsenite Removal by Zerovalent Iron: Kinetics, Redox Transformation, and Implications for in Situ Groundwater Remediation. *Environmental Science & Technology* **35**, 1487–1492 (2001).

6. Georgiou, Y. *et al.* Surface decoration of amine-rich carbon nitride with iron nanoparticles for arsenite (As III) uptake: The evolution of the Fe-phases under ambient conditions. *Journal of hazardous materials* **312**, 243–253 (2016).
